# Evaluating Childhood Overweight- and Obesity-Related Food Marketing Policies in China Using the Food–Environment Policy Index (Food–EPI)

**DOI:** 10.3390/nu16040482

**Published:** 2024-02-07

**Authors:** Zhenhui Li, Yujie Fang, Na Zhang, Wenli Zhu, Suying Chang, Shuyi Zhou, Man Zhang, Guansheng Ma

**Affiliations:** 1 Department of Nutrition and Food Hygiene, School of Public Health, Peking University, 38 Xue Yuan Road, Haidian District, Beijing 100191, China; lizhenhui@bjmu.edu.cn (Z.L.); fangyujie@pku.edu.cn (Y.F.); zhangna@bjmu.edu.cn (N.Z.); zhuwenli@bjmu.edu.cn (W.Z.); 2211110217@stu.pku.edu.cn (S.Z.); 2 Laboratory of Toxicological Research and Risk Assessment for Food Safety, Peking University, 38 Xue Yuan Road, Haidian District, Beijing 100191, China; 3 Child Health Development Section, United Nations International Children’s Emergency Fund (UNICEF) Office for China, 12 Sanlitun Lu, Chaoyang District, Beijing 100600, China; schang@unicef.org; 4 School of Nursing, Peking University, 38 Xue Yuan Road, Haidian District, Beijing 100191, China

**Keywords:** food marketing, Food–Environment Policy Index, childhood overweight and obesity, policy, China

## Abstract

Objective: Addressing the increasing global health issue of childhood obesity, exacerbated by pervasive food marketing, this study critically evaluated China’s food marketing policies in comparison with international best practices, aiming to uncover policy content and implementation gaps and inform policy enhancement strategies. Method: Three key indicators were utilized from the Healthy Food–Environment Policy Index (Food–EPI)’s food promotion domain. A panel of experts (*n* = 13) from academic institutions, China Centers for Disease Control and Prevention, and the food industry assessed the Chinese government’s policy scores and implementation levels concerning food marketing. Benchmarked against international best practices using the Food–EPI process, this evaluation encompassed context analysis, data collection, evidence-based policy action, government validation, policy rating, scoring, and results translation for government and stakeholders. The three chosen indicators specifically addressed childhood overweight- and obesity-related food marketing in broadcast media (Indicator 1), non-broadcast media (Indicator 2), and child gathering settings (Indicator 3). Results: Specifically, Indicator 1, the Single Food Marketing Indicator Score was measured at 2.31 ± 0.38, with an accompanying Food Marketing Policy Implementation Percentage of 46.2%, and Low Implementation Level. For non-broadcast mediums (Indicator 2), these metrics were gauged at 1.77 ± 0.27, 35.4%, and Low Implementation Level, respectively. In child gathering settings (Indicator 3), for efforts curbing unhealthy food promotion, a score of 2.77 ± 0.27, an implementation percentage of 55.4%, and Medium Implementation Level was obtained. Cumulatively, the overarching efficacy of food marketing policy enforcement was determined to be suboptimal, with the consolidated figures being Total Food Marketing Score as 2.28 ± 0.97, Total Food Marketing Policy Implementation Percentage as 45.6%, and Total Food Marketing Policy Implementation Level as Low. Conclusion: Like many countries, China’s food marketing policies and implementation have room for improvement when compared to international best practices. Recommendations include emphasizing nutritional legislation, fostering stakeholder collaboration, bolstering public health campaigns, and leveraging technology for stringent enforcement.

## 1. Introduction

Childhood obesity and related non-communicable diseases (NCDs) have become increasingly prevalent global public health issues, notably in China. The National Survey on Student Physical Fitness and Health revealed a rise in China’s childhood obesity rates from 1.24% in 1985 to 27.97% in 2019 [1]. Without further effective interventions, by 2030, 60% of Chinese children will be affected by overweight and obesity [2]. The economic burden linked to childhood obesity may cost CNY 218 trillion annually in the next 60 years [2]. Direct and indirect economic losses from childhood obesity may account for CNY 261 billion and CNY 217 trillion CNY, respectively [2]. These trends suggest significant future health challenges, potentially leading to diseases like type 2 diabetes, cardiovascular disorders, and certain cancers.

The global consensus identifies the food environment as one of a key driver of childhood overweight and obesity. Within this context, food marketing, a core element of the food environment, has significantly influenced children’s dietary choices, eating behavior, and lifestyle. Although food marketing has efficiently connected manufacturers and consumers, introducing innovative products and stimulating market dynamics, its impact on children’s public health is substantial and warrants careful consideration [3,4].

Contemporary food marketing strategies, replete with sophisticated digital mediums and vibrant visuals, have exerted an insidious influence on children, shaping their dietary choices [4,5]. Given their developing cognitive abilities, children are often have remained oblivious to the strategic intents embedded within these promotions, rendering them prime targets for advertisers. This paradigm, accentuated by colorful imagery and compelling mascots, subtly steers children towards high-caloric, nutrient-deficient foods. The confluence of such marketing tactics with children’s susceptibilities invariably amplifies the obesity trajectory, setting the stage for a gamut of health complications, ranging from premature weight gain to chronic diseases [6,7,8].

Countries worldwide have adopted varied policies to counter food marketing targeting children. Common strategies include limiting unhealthy food advertisements during children’s TV hours, as seen in Norway, Sweden, and Ireland [9,10]. Regions like Quebec and Chile also restrict such marketing on digital and social media [10]. Efforts extend to schools, with countries like Mexico banning unhealthy food advertisements on campuses [11]. Public campaigns and educational initiatives are aimed at enhancing nutritional literacy, empowering children and their families to make informed food choices amidst pervasive advertising.

China has initiated a range of policies to mitigate the impact of food marketing on childhood obesity, reflecting a strategic commitment to integrating health considerations within its food policy framework. Yet, a critical gap remains—these policies have not been systematically analyzed for their alignment and comparation with international standards. Hence, our study underscores the necessity for using a robust framework to systematically review, analyze, and evaluate these policies.

The Healthy Food–Environment Policy Index (Food–EPI), devised by the International Network for Food and Obesity/Non-communicable Diseases Research, Monitoring and Action Support (INFORMAS) in 2013 [12], served as a framework for governments to evaluate and improve food environments. Implemented first in New Zealand in 2014, it has since been embraced by around 30 nations. Demonstrating commendable inter-rater reliability (GwetAC2 = 0.6–0.8), the Food–EPI stands as a reliable and adaptable tool for gauging the effectiveness of food-related policies across various national contexts [10].

Therefore, this manuscript utilized three indicators form the Food–EPI to critically appraise China’s current childhood overweight- and obesity-related food marketing policy framework. By undertaking a comparative analysis with international best practices, our objective was to furnish pragmatic recommendations, refining China’s policy landscape to foster a more health-promoting food marketing environment for its citizenry.

## 2. Materials and Methods

**Figure 1:**
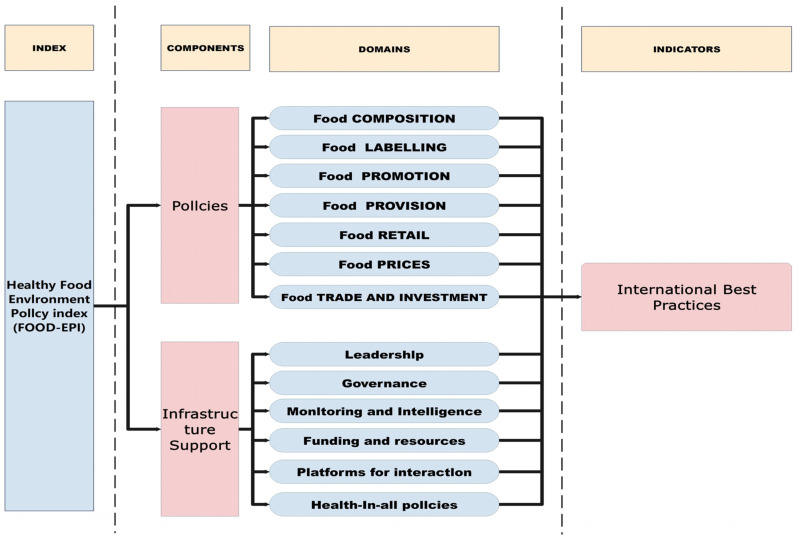
Components and domains of the Healthy Food–Environment Policy Index (Food–EPI).

The Food–EPI encompasses over 40 indicators spanning seven food policy domains and six structural dimensions, which are shown in Figure 1 [12]. Each of the indicators includes several international best practices to make it easier for researchers to understand the gaps between their national policies and internationally recommended policies.

In the academic literature, distinctions between ‘food marketing’ and ‘food promotion’ are evident, though the terms are occasionally used interchangeably in Food–EPI studies [13,14]. While ‘food marketing’ encompasses a wide spectrum, including product development and distribution, ‘food promotion’ specifically zeroes in on strategies amplifying product visibility, notably through media and educational avenues. This paper is geared towards assessing the latter’s influence on childhood overweight and obesity.

However, our choice to employ ‘food marketing’ over the more specific ‘food promotion’ is principally influenced by linguistic and conceptual understandings in the Chinese context, the primary milieu of our research. In Chinese discourse, ‘food marketing’ resonates more adeptly, making it a more apt term for our study’s purpose and audience. Thus, while our focus aligns closely with ‘food promotion’, we opt for the term ‘food marketing’ to maintain cultural and linguistic relevance.

**Figure 2:**
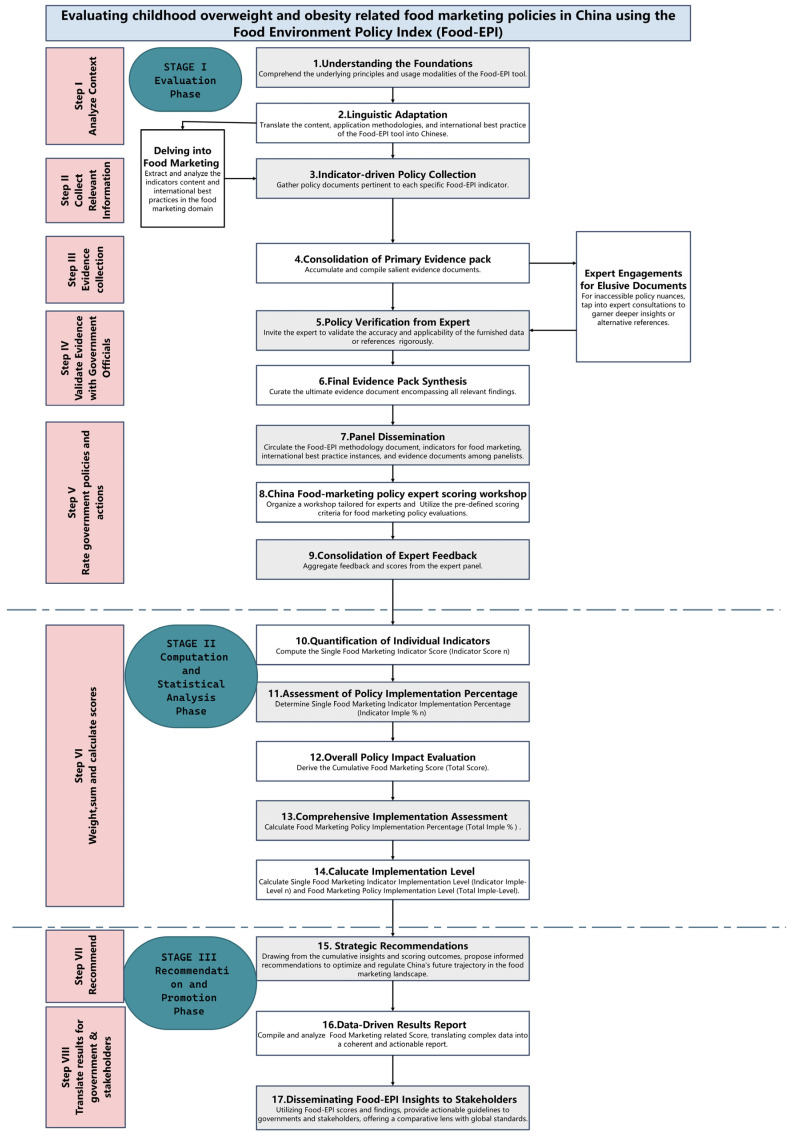
Evaluating food-marketing-related policies in China using the Food–Environment Policy Index (Food–EPI).

To critically appraise the design of food marketing policies in China, we derived 3 metrics including the Toal Food Marketing Score (*Total Score*), the Total Food Marketing Policy Implementation Percentage (*Total Impl* %), and the Total Food Marketing Policy Implementation Level (*Total Impl-Level*). Our evaluative framework and calculation process draws upon the octadic structure of the Food–EPI. For clarity and comprehensive understanding, we further demarcated these eight steps into three well-defined phases, encompassing a total of 17 distinct tasks, as shown in Figure 2.

### 2.1. Step I: Analyze Context 

#### 2.1.1. Understanding the Foundations

Following a literature review, we determined the Food–EPI’s principles and operational methods in food environment policy evaluation. We chose all three indicators in the food marketing domain from the Food–EPI to scrutinize childhood overweight- and obesity-related food marketing policies in China (as shown in Table 1). For clarity and ease of reference in the subsequent discussion, we refer to ‘childhood overweight- and obesity-related food marketing policy’ simply as ‘food marketing policy’.

#### 2.1.2. Linguistic Adaptation

To assist experts new to the Food–EPI framework, we translated its methodology, three key indicators, and international best practice examples into Chinese. This translation was vital for providing a familiar context, and to enhance the understanding of the framework’s purpose and procedures. The task, initially executed by a primary researcher, was rigorously validated by two others to ensure accuracy and efficacy in subsequent evaluations.

### 2.2. Step II: Collect Relevant Information

#### Indicator-Driven Policy Collection

An exhaustive review of policy documents addressing food marketing in China from January 2000 to September 2023 was undertaken. This encompassed laws, national and partly provincial level regulation, guidelines, standards, and actions (Table 2). 

For comprehensive data collection pertaining to the Food–EPI framework, we partly adopted a structured, multi-tiered approach, delineated by Amos Laar et al. in their assessment of Ghana’s food environment [15], as shown in Box 1.

During the policy document collection, we reviewed different policies targeting food marketing potentially influencing childhood overweight and obesity. This included policies across broadcast and non-broadcast platforms and school settings, encompassing a wide range of food products. Notably, our collection extended to regulations governing the marketing of infant formula. This inclusion reflected a recognition of the critical impact early food intake, particularly concerning infant formula, can have on long-term health outcomes and obesity trajectories in children [16,17,18,19,20]. We aimed to include policies affecting all food types consumed by children from birth, ensuring a complete view of food marketing policy in China.

Box 1. **China Food marketing policy evaluation-specific steps for identifying policy evidence.**

Step One: Leveraging stakeholder mapping, we pinpointed essential pub-lic/government organizations integral to 3 food marketing indicators. Renowned portals such as the Chinese Government’s official website and the National Health Commission served as primary sources.

Step Two: We systematically scoured these identified organizational web-sites for pertinent policy or infrastructure evidence, cataloging findings via a spe-cialized online shared form (Tencent Documents) and aligning them with relevant Food–EPI domains/indicators. Law repositories like PKU-Law were used in this phase.

Step Three: In instances where specific organizational websites weren't iden-tified, or post the exhaustive website mining, we directly engaged with these key organizations. Through focused discussions, we sought clarity on extant evidence related to different policy and support domains. Recognized NGOs and prominent search engines further enriched this phase of data collation.

Step Four: Upon the identification of salient policies or initiatives, a more concentrated search was embarked upon. Tapping into academic databases, like CNKI, PubMed, Web of Science, and Springe, we utilized specific key terms associ-ated with these discerned policies/initiatives, ensuring a thorough and nuanced understanding of each.

### 2.3. Step III: Evidence collection

#### Consolidation of Primary Evidence Pack

With the completion of the data collection, policy documents were systematically collated into an initial draft evidence file. Each entry in this draft was meticulously detailed, encompassing the policy’s name, release date, issuing agency, hierarchical policy level, and the specific content related to the intended indicators.

### 2.4. Step IV: Validate Evidence with Government Officials

#### 2.4.1. Policy Verification from Experts

To validate the fidelity and comprehensiveness of the primary evidence pack, ten Chinese experts and relevant government functionaries were enlisted. Experts were identified and invited via the authors’ networks. The criteria included having at least 10 years of experience in related fields, like childhood obesity prevention or food marketing, and holding a professorship (or equivalent) title or above. Government employees with experience in childhood obesity prevention policy formulation were also involved. Employing online consultations, this validation ensured the document’s rigor and pertinence. We pursued two rounds of online consultations. 

An essential facet of the research was comparing local policies against international best practices. These “benchmark” standards were obtained from the pivotal “Benchmarking Food Environments 2017” document published by the INFORMAS Secretariat [10]. After discussion, we chose not to adjust and modify these practices.

#### 2.4.2. Final Evidence Pack Synthesis

Based on feedback from the two rounds of online consultations, we refined our evidence pack. This pack includes details of each indicator’s definition, international best practices, and collation of relevant Chinese policies. The evidence pack provided a pivotal foundation to facilitate in-depth evaluation of China’s food marketing policy landscape.

### 2.5. Step V: Rate Government Policies and Actions 

#### 2.5.1. Panel Dissemination

To rate China’s food marketing policies, we convened an enlarged panel of 13 experts. The criteria used remained consistent with the Policy Verification from the “Expert” phase. We first invited all 10 experts from last step via email and subsequently broadened our panel by inviting additional specialists. This expansion sought to ensure a wider array of expert viewpoints, thereby enhancing the panel’s representativeness and stakeholder inclusivity.

To prepare the expert participants for the rating workshop in the next step, we distributed key documents, including the Food–EPI methodology (in both Chinese and English), the Final Evidence Pack, and the rating criteria, two weeks in advance. This preparation aimed at ensuring informed and comprehensive evaluation by the panel.

#### 2.5.2. China Food Marketing Policy Expert Scoring Workshop

Hosted within a dedicated conference setting, all 13 experts of the panel attended the workshop. Each indicator’s assessment was systematically introduced through a bifocal presentation approach: the initial slide delineated the policies instituted by China, while the succeeding one juxtaposed these frameworks against prevailing international best practices. Following this, the experts, equipped with a purpose-designed scoring table (see Appendix A), provided their evaluative ratings and suggestions in adherence to predefined criteria (show in Table 3). 

#### 2.5.3. Consolidation of Expert Feedback

After the workshop, we focused on processing the expert feedback. First, a researcher input the ratings and suggestions from the scoring table into a computer database. Then, to ensure accuracy, two other researchers double-checked the data.

### 2.6. Step VI: Weight, Sum, and Calculate Scores

The synthesis of scores from our experts resulted in a *Total Score*, *Total Impl %* and *Total Impl-Level*. Descriptive statistics were generated using Microsoft 365MSO (Version 2401). The computation process is elucidated below. 

#### 2.6.1. Quantification of Individual Indicators

For each food marketing indicator, the Single Food Marketing Indicator Score (*Indicator Score n*, full points is 5) is equal to the average score of the experts (*Expert m*) for this indicator:

$$Indicator\;Score\;n=\frac{\sum (Expert1+Expert2+Expert3+\ldots +Expertm)}{m}\;(n\;\mathrm{equal}\;\mathrm{to}\;1,\;2,\;3).$$

#### 2.6.2. Assessment of Policy Implementation Percentage

The *Indicator Score n* was used to calculate the Single Food Marketing Policy Implementation percentage (*Indicator Impl % n*) (“Food Market Policy Implementation percentage” refers to the extent to which a country or region has implemented or adopted recommended food marketing policies relative to a set of international best practices. It tells us how close a country or region is to fully implementing best practice related to food marketing. The higher the percentage, the closer the country or region is to the ideal or recommended standard):

$$Indicator\;Impl\;\%\;n=\frac{Indicator\;Score\;n}{5}\times 100\%\;(n\;\mathrm{equal}\;\mathrm{to}\;1,\;2,\;3)$$

#### 2.6.3. Overall Policy Impact Evaluation

Each indicator’s weight is the same, equal to 1. Thus, the *Total Score* (full points is 5) is equal to the average of the three indicators, i.e.,

$$Total\;Score=\frac{\sum (Indicator\;Score\;1+Indicator\;Score\;2+Indicator\;Score\;3)}{3}$$

#### 2.6.4. Comprehensive Implementation Assessment

The *Total Impl %* equal to the *Total Score* divided by 5 points (full points for *Total Score*) and times 100%:

$$Total\;Impl\;\%=\frac{Total\;Score}{5}\times 100\%$$

#### 2.6.5. Calculation of the Implementation Level

Based on the Food–EPI, the *Indicator Impl % n* and the *Total Impl %*were then stratified into different indicator’s implementation levels (*Indicator Impl-Level n*) and Total Food Marketing Policy Implementation Level (*Total Impl-Level*) as follows: ≥75% as ‘High’, 50–75% as ‘Medium’, 25–50% as ‘Low’, and <25% as ‘Very Low’ (for example, if the *Indicator Score 1* (the first indicator’s score) is 2.5, *Indicator Impl % 1* and *Indicator Impl-Level 1* will be 50% and Medium, respectively).

### 2.7. Step VII: Recommendations

#### Strategic Recommendations

Considering the calculated scores and the expert feedback, we formulated strategic recommendations to refine China’s food marketing policies. Our approach centered on identifying indicators with lower scores and integrating expert insights on prospective policy improvements. This enabled us to pinpoint specific domains requiring attention and to propose targeted strategies which would align China’s policies more closely with global best practices and expert perspectives on future enhancements in food marketing.

### 2.8. Step VIII: Translate Results for Government and Stakeholders

#### 2.8.1. Data-Driven Results Report

Our analysis provides a comprehensive view of China’s food marketing policy landscape, presenting data with graphical enhancements for clarity and comparability with international best practices. This concise overview highlights key findings and offers strategic insights for future policy development in China’s food marketing sector.

#### 2.8.2. Disseminating Food–EPI Insights to Stakeholders

Moving forward, the research outcomes of our study on China’s food marketing policies will be shared with key stakeholders, including government entities. Through academic forums and policy discussions, we aim to foster informed collaboration, focusing on refining and implementing effective policies to address childhood overweight and obesity linked to food marketing.

## 3. Results

### 3.1. Characteristics of Evidence on Government Policy Action on Food Marketing in China

Based on an exhaustive policy scan, we selected 10 policy documents that met specific criteria in the food marketing domain, which are shown in Table 4 below. 

### 3.2. Characteristics of Local Expert Panel

All 13 invited experts attended the rating workshop. The panel, comprising 7 males and 6 females, spanned disciplines from nutrition and food hygiene (*n* = 4) to public health (*n* = 2), marketing (*n* = 2), public policy (*n* = 2), clinical medicine, governmental administration, and communication studies. Regarding their organization, the 13 experts were from academic institutions (*n* = 7), the CDC (*n* = 5), and the food industry. All 10 experts involved in the evidence pack review continued their involvement in the scoring workshop, leveraging their project expertise in the project.

### 3.3. Score, Implementation Percentage, and Implementation Level in Food Marketing

Statistical analyses undertaken determined the Toal Food Marketing Score (*Total Score*) to be 2.28, with a 95% confidence interval (CI) of 1.31 to 3.25. This quantification translated to a Total Food Marketing Policy Implementation Precent of 45.6% (*Total Impl % = 45.6%*), categorized as a low level of implementation (*Total Impl Level =* Low). Delving into the granular details, the individual indicator scores, and the corresponding implementation percentages and the implementation level for the triad of indicators were as follows: 2.31 (95% CI: 1.93, 2.69, 46.2%, Low), 1.77 (95% CI: 1.50, 2.04, 35.4%, Low), and 2.77 (95% CI: 2.50, 3.04, 55.4%, Medium), as shown in Table 5. 

### 3.4. Food Marketing Indicator 1

#### 3.4.1. Local Evidence

China’s stringent regulations, complemented by industry standards, rigorously curb unhealthy food advertisements targeting children, especially those advocating use of mother’s milk substitutes, representing a comprehensive approach to protecting children’s nutritional well-being (show on Table 6).

#### 3.4.2. International Best Practices

See Appendix B.

### 3.5. Food Marketing Indicator 2

#### 3.5.1. Local Evidence

Despite the absence of a law in China on non-broadcast unhealthy food promotions for minors, the “National Program for the Development of Children” has delineated clear regulations for child-focused media channels. Additionally, regions like Shenzhen introduced targeted measures, such as health warnings on selected beverages, reinforcing a commitment to safeguard children from adverse food marketing (see Table 7).

#### 3.5.2. International Best Practices

See Appendix C.

### 3.6. Food Marketing Indicator 3

#### 3.6.1. Local Evidence

China’s legal frameworks stringently curtail commercial food marketing in educational settings and emphasize the prohibition of unhealthy products (see Table 8).

#### 3.6.2. International Best Practices

See Appendix D.

## 4. Discussion

### 4.1. Significance of the Study

This study represents the first comprehensive evaluation of China’s food marketing policies in the context of rising child obesity. It involved a comparative analysis of China’s childhood obesity-related food marketing policies against international best practices, focusing on their content and execution effectiveness. In a globalized context where international marketing strategies influence local trends, this research is crucial for aligning China’s policies with global practices, offering vital insights for stakeholders, academics, and policymakers.

### 4.2. Comparative Analysis with International Best Practices

In the complex realm of global public health, particularly childhood obesity prevention, contextualizing China’s food marketing policies vis-à-vis international benchmarks has become paramount.

#### 4.2.1. Alignment with International Best Practices

China’s strategies revealed noteworthy alignments with international paradigms. Restrictions on infant formula marketing and the prohibition of unhealthy food promotion within educational environments resonate with global standards, underscoring China’s commitment to mitigate the detrimental impact of food marketing on children, as highlighted in significant official documents.

#### 4.2.2. Areas of Discrepancy

China’s approach to regulating food marketing, particularly in non-broadcast spheres like online shopping websites, social media, and short video platforms, contains gaps when compared to the more comprehensive measures implemented in countries like Canada and Chile. This shortfall in addressing non-broadcast food marketing, especially concerning content that could contribute to childhood obesity, is not unique to China but reflects a global trend. The significance of managing food marketing in online media has only recently been recognized globally, leading to a widespread realization of its impact on childhood health. This underscores a common challenge faced by many countries in adapting to the evolving landscape of digital media and its influence on public health.

#### 4.2.3. A Global Conundrum—Policy Implementation

An overarching theme emerges when analyzing China’s policy efforts alongside global counterparts, that of the persistent challenge of policy enactment. Based on the availability of comparable data and use of a consistent methodology, notwithstanding the articulation of robust strategies, the global community, including China, faces the challenge of ensuring effective policy realization (Table 9 and Table 10). The recurrent gaps between policy formulation and its operationalization underscore a shared global imperative to address these.

### 4.3. Implications of Low and Moderate Scoring

In the landscape of China’s child-centric food marketing, suboptimal regulatory adherence has resulted in profound health, economic, and societal repercussions. Pediatric vulnerability to aggressive marketing amplifies risks for early onset obesity, potentially contributing to the development of chronic diseases like type 2 diabetes in later life. This trajectory imposes a dual economic strain, including both immediate health costs and forecast productivity losses amounting to a staggering USD 49.02 billion by 2050 [25]. Additionally, familial dynamics are strained by the repercussions of obesity, with burgeoning health disparities potentially aggravating societal inequalities, especially among socioeconomically disadvantaged populations [26,27,28,29,30,31].

### 4.4. Limitations of the Study: Navigating the Intricacies of Research and Interpretation

There are two limitations to our study. Firstly, while the expertise of our panel adds credibility, their subjective interpretations, cognitive differences, and personal experiences could influence their ratings. Secondly, due to variations in data availability, modifications in indicators, and methodological disparities, certain countries’ results were not included in our study, affecting the comparative analysis between China and other nations. 

## 5. Policy Implication and Suggestion

Addressing childhood overweight and obesity in China demands an integrated strategy. Informed by our meticulous research, we offer the following innovative and pragmatic solutions:

### 5.1. Foundational Nutritional Legislation

China is at an early stage of development in terms of establishing nutrition-specific legal edicts. The initiation of a concrete legal framework for food marketing, aligned with ‘Healthy China 2030’, would affirm a national health commitment and represent a transformative moment in nutritional governance.

### 5.2. Integrated Stakeholder Engagement via COM and PPPs

To ensure policy efficacy, establishing a centralized oversight mechanism (COM) is crucial. This mechanism would integrate diverse sectoral perspectives, optimizing policies to mitigate food marketing’s effects on children. Additionally, fostering the use of public–private partnerships (PPPs) can enable benefit from industry insights, helping to align market strategies with child health priorities and to balance profit motives with public health needs.

### 5.3. Amplified Public Health Literacy through Strategic Campaigns

In today’s digital era, shaping perceptions is key. Curated health campaigns, leveraging media platforms frequented by the youth, can foster a discerning attitude towards food marketing. By spotlighting the implications of unhealthy dietary choices and demystifying marketing tactics, such initiatives can help create a populace that is more resistant to misleading advertisements and more adept at making informed nutritional decisions.

### 5.4. Technologically Enhanced Enforcement

The crux of policy efficacy lies in its vigilant enforcement. Investing in cutting-edge surveillance systems, augmented with AI capabilities, can ensure consistent adherence. Additionally, crafting comprehensive guidelines tailored for digital communication can help guarantee marketing transparency, preventing any obfuscation of nutritional facts.

## 6. Conclusions

In comparison to international best practices, China’s existing food marketing policies demonstrate room for improvement in terms of both content and implementation, reflecting a common global issue. To narrow the gaps and to elevate policy efficacy, enhancing nutritional legislation, encouraging collaboration among stakeholders, intensifying public health awareness campaigns, and employing advanced technology are suggested.

## Figures and Tables

**Table 1:** Food marketing and each indicator context.

Domain/Indicator	Content
Food Marketing	There is a comprehensive regulatory approach implemented to reduce the impact (exposure and power) of the promotion of unhealthy foods and beverages (high in saturated fats, trans-fats, added sugars and/or salt) to children (e.g., <16 years) across all media.
Indicator 1 (Broadcast Media Restrictions)	Effective policies are implemented by the government to restrict exposure and the power of promotion of unhealthy foods to or for children through broadcast media (TV, radio)
Indicator 2 (Non-Broadcast Media Restrictions)	Effective policies are implemented by the government to restrict exposure and the power of promotion of unhealthy foods to children through non-broadcast media (e.g., Internet, social media, food packaging, sponsorship, outdoor advertising, including around schools)
Indicator 3 (Child Gathering Settings)	Effective policies are implemented by the government to ensure that unhealthy foods are not commercially promoted to children in settings where children gather (e.g., preschools, schools, sport, and cultural events)

**Table 2:** Policy document types and definitions.

Type	Issuing Authority	Definition and Features	Mandatory
Law	National People’s Congress and its Standing Committee	Formal statutory norms crafted, amended, and promulgated with state-enforced powers.	Strongest
Regulation	State Council, subordinate departments, local governments	Non-legislative directives aiding the realization of legal principles.	Varies significantly based on issuing department
Guidelines	Any individual or institution	Advisory opinions and recommendations for specific domains.	Low to Medium
Standards	National, industry, regional, group, enterprises, etc.	Rules or characteristic values that can be mandatory or voluntary. Includes national, industrial, and other standards.	Variable; can be mandatory or voluntary
Action Plan	Various (depending on the specific action)	Specific plan for activities and measures undertaken to realize and implement laws and policies.	Dependent on specific action and context

**Table 3:** Scoring criteria.

Score	Criteria	Policy Alignment with Best Practices (Note: The “Alignment” Column Signifies the Extent to Which Domestic Policies Adhere to Recognized International Best Practices)
1	Absence or nominal policy presence	<20%
2	Rudimentary policy structures	20–40%
3	Intermediate policy implementation	40–60%
4	Robust policy strategies	60–80%
5	Comprehensive and holistic policy framework	>80%

**Table 4:** Policy or related documents regarding the three food marketing indicators.

Food Marketing Indicator	Name	Date of Issue	Agency	Type	Mandatory Level
Indicator 1 (Broadcast Media Restrictions)	Advertisement law of the People’s Republic of China	29 April 2021	Standing Committee of the National People’s Congress	Law (National)	High
	Self-regulation rules for infant formula advertising	27 October 2019	China Advertising Association	Standard (Industry Standard)	Low
Indicator 2 (Non-Broadcast Media Restrictions)	National Program for the Development of Children	8 September 2021	The State Council of the People’s Republic of China	Regulations (National)	Medium
	Health regulations of the Shenzhen special economic zone	24 November 2020	Health Commission of Shenzhen	Regulations (Special economic zone)	Medium
Indicator 3 (Child Gathering Settings)	Advertisement Law of the People’s Republic of China	29 April 2021	Standing Committee of the National People’s Congress	Law (National)	High
	Law of the People’s Republic of China on the protection of minors	17 October 2020	Standing Committee of the National People’s Congress	Law (National)	High
	National Program for the Development of Children	8 September 2021	The State Council of the People’s Republic of China	Regulations (National)	Medium
	Guidelines for building nutrition and health schools	7 June 2021	Ministry of Education State Administration for Market Regulation National Health Commission General Administration of Sport	Guideline (National)	Medium
	Opinions on Comprehensively Strengthening Food Safety Supervision (Ji Ling Province)	23 April 2019	CPC Jilin Provincial Committee Jilin Provincial People’s Government	Regulations (Province)	Medium
	Regulations on School Food Safety, Nutrition and Health Management	20 February 2019	Ministry of Education State Administration for Market Regulation National Health Commission	Regulations (National)	Medium
	Action Plan for Oral Health (2019–2025)	16 February 2019	National Health Commission	Action Plan (National)	Medium
	Notice on Further Strengthening School Food Safety and Promoting a Balanced Diet (Guangdong Province)	13 December 2015	Department of Education of Guangdong Province Guangdong Provincial Market Supervision Administration, Health Commission of Guangdong Province	Regulations (Province)	Medium

**Table 5:** Score, implementation percentage, and implementation level for Food Marketing.

Domain/Indicator	Food Marketing Policy Indicator Score	Food Marketing Policy Implementation Percentage	Food Marketing Policy Implementation Levels
Food Marketing (Total)	2.28 ± 0.97	45.6%	
Indicator 1 (Broadcast Media Restrictions)	2.31 ± 0.38	46.2%	
Indicator 2 (Non-Broadcast Media Restrictions)	1.77 ± 0.27	35.4%	
Indicator 3 (Child Gathering Settings)	2.77 ± 0.27	55.4%	
		Low	
		Medium	

**Table 6:** Local evidence on Food Marketing Indicator 1.

File Type	Content
Law	The *Advertisement Law of the People’s Republic of China* states that advertisements advocating replacing mother’s milk with infant milk products, beverages, and other foods are prohibited. Using minors under ten years old as advertising spokespeople is also prohibited. Violations will result in penalties ranging from one to three times the advertising costs or fines ranging from CNY 100,000 to CNY 1,000,000 (USD 15,000 to USD 150,000).
Regulations (National)	The *National Program for the Development of Children* delineates stringent guidelines pertaining to child-centric food marketing. Broadcasts targeting children are prohibited from showcasing advertisements related to pharmaceuticals, health foods, alcoholic beverages, and especially those suggesting alternatives to breast milk. Emphasizing the well-being of children, the program restricts misleading and deleterious advertising content while bolstering efforts to penalize non-compliant practices. Furthermore, regulations curtail children’s involvement in commercial promotional activities.
Industry Standards (Not mandatory)	In 2016, the China Advertising Association promulgated the “*Self-regulation Rules for Infant Formula Advertising*.” Central to its provisions, Article 5 mandates clear distinctions between imagery for “infant” and “older infant” formulas. Advertisements are prohibited from misrepresenting formulas as being analogous to breastmilk. To ensure compliance, the association enforces corrective advisories and, for recalcitrant or egregious offenders, issues public censures.

**Table 7:** Local evidence on Food Marketing Indicator 2.

File Type	Content
Law (National)	Currently, there are no national laws limiting the exposure and promotion of unhealthy foods to children through non-broadcast mediums.
Regulations (National)	The “*National Program for the Development of Children*” rigorously prescribes non-broadcast marketing standards for child-centric products and services. Specifically, it proscribes advertisements for health, beauty, and alcohol-related products, alongside potentially harmful online games, in media channels tailored for children. A stringent prohibition is enforced on promotions in the mass media that present any food items as replacements for breast milk. To bolster these mandates, the program has intensified its oversight, ensuring punitive measures are taken against deceptive and illicit advertisements.
Regulations (Special economic zone)	On 29 October 2020, the forty-fifth meeting of the Standing Committee of the Sixth People’s Congress of Shenzhen City passed *the Health Regulations of the Shenzhen Special Economic Zone*, which will take effect on 1 January 2021. Article 47 states “The sellers of alcoholic beverages and carbonated beverages shall set up health damage warning signs on the shelves or counters that meet the standards. The production standards and setting norms of health damage warning labels for alcoholic beverages and carbonated beverages shall be formulated by the municipal health department and announced to the public”

**Table 8:** Local evidence on Food Marketing Indicator 3.

File Type	Content
Law (National)	Chinese laws strictly regulate the advertising environment within educational settings. According to the *Advertisement Law of the People’s Republic of China*, advertising activities are prohibited in primary and secondary schools as well as kindergartens. The law further restricts the use of school-related items, like educational materials, school uniforms, and school buses, for advertising purposes, except for public service advertisements. Similarly, the *Law of the People’s Republic of China on the Protection of Minors* bans any advertisement that could be harmful to the physical or mental well-being of minors and disallows the distribution of commercial advertisements in educational institutions.
Regulations (National)	In 2019, a collaborative regulation titled “*Regulations on School Food Safety and Nutritional Health Management*” was released by China’s Ministry of Education, State Administration for Market Regulation, and the National Health Commission. This directive concerned the restricted establishment of food retail venues within educational institutions, emphasizing reduction in high salt, sugar, or fat foods.
Regulations (Province)	The Education Department of Guangdong Province, in 2015, issued the “*Notice on Further Strengthening School Food Safety and Promoting Dietary Nutritional Balance*,” which provided guidance on refraining from the sale of products detrimental to health, such as soda and instant noodles, within school premises, emphasizing youth health protection. In April 2019, Jilin Provincial Committee of the Communist Party of China and the Jilin Provincial Government Office published the “*Opinions on Comprehensively Strengthening Food Safety Supervision*”, which advocates for strong regulation of food safety, especially within educational environments, targeting venues like school canteens and certain snack items
Guideline (Mandatory)	On 7 June 2021, China’s Ministry of Education, in collaboration with the National Health Commission and other departments, issued the “*Guidelines for the Construction of Nutrition and Health in Schools*.” These guidelines prohibit the sale of high salt, sugar, or fat foods, and alcoholic beverages within primary and secondary schools. Specifically, Article 25 of the guidelines restricts the establishment of food retail venues within schools and bans the advertisement of sugary drinks and seasoned snack products. While the guidelines primarily target full-time regular primary and secondary schools, they also suggest that tertiary institutions, vocational schools, and kindergartens may refer to these norms when promoting nutrition and health.
Action Plan	The National Health Commission’s “*Action Plan for Oral Health (2019–2025)*” underscores the imperative of a strategic “sugar reduction” initiative. Integral to this strategy, primary and secondary educational establishments, alongside early childhood institutions, are encouraged to curtail the distribution of high-sugar beverages and snacks. Concurrently, there is an advisory for educational institution canteens to diminish the provision of beverages and foods with elevated sugar content.

**Table 9:** Total Food Marketing Policy enactment percentage in different countries or regions.

China	Canada	Estonia	EU	Finland	Germany	Ghana
Low	Low	Low	Low	Medium	Very Low	Low
Ireland	Italy	Kenya	Netherland	New Zealand	Norway	Poland
Low	Low	Low	Medium	Low	Low	Medium
Portugal	Slovenia	Spain				
Medium	Medium	Low				

Data from Vanderlee et al., 2019 [21], Asiki et al, 2019 [22], Vandevijvere et al., 2018 [23], and Laar et al., 2020 [15].

**Table 10:** Single Food Marketing Indicator Policy enactment percentage in different countries or regions.

Domain/Indicator	Food Marketing Indicator 1	Food Marketing Indicator 2	Food Marketing Indicator 3
China	Low	Low	Moderate
Eu (Pineda et al., 2022 [14])	Low	Low	Low
Canada (Vanderlee et al., 2019 [21])	Low	Low	Low
Ghana (Laar et al., 2020 [15]; Wang et al., 2020 [24])	Low	Low	Low
Kenya (Asiki et al., 2019 [22])	Low	Low	Low
New Zealand (Vandevijvere et al., 2018 [23])	Low	Low	Low

## Data Availability

Data is contained within the article.

## Appendix A

**Table A1:** Childhood Overweight- and Obesity-Related Food Marketing Policies in China Evaluation Rating Scale.

Name	
Index Content	**Indicator 1**: Effective policies are implemented by the government to restrict exposure and the power of promotion of unhealthy foods to or for children through broadcast media (TV, radio).
International Best Practices	Norway/Sweden: Per the Broadcasting Act, advertisements, irrespective of food content, are prohibited from being broadcast during or in association with children’s programs for those aged 12 and under.
	Quebec (Canada): In Quebec, the only Canadian province to implement such measures since 1980, the Consumer Protection Act strictly proscribes commercial advertisements targeting children under 13 across various media. This determination considers the advert’s content, presentation, and the broadcasting context. A threshold of 15% child viewership is set for protective measures against TV marketing. Violation of this statute at any commercial stage incurs significant penalties, ranging from CAD 600 to CAD 15,000 for individuals and CAD 2000 to CAD 100,000 for entities. Conversely, the rest of Canada relies on self-regulation, operationalized through the Canadian Children’s Food and Beverage Advertising Initiative (CAI) and enforced by Advertising Standards Canada (ASC) via The Broadcast Code for Advertising to Children.
	Ireland: In line with the 2013 revision of the Children’s Commercial Communications Code, advertisements of foods high in fats, sugars, and salt—as determined by a nutrient profiling model—are restricted during children’s TV and radio broadcasts with over 50% under-18 audience. Additionally, these foods are capped at 25% of total advertising time, with one in every four advertisements permissible. Any remaining adverts aimed at those below 13 years cannot employ health claims or licensed characters.
	South Korea: TV advertising to children less than 18 years of age is prohibited for specific categories of food before, during, and after programs shown between 5 and 7 pm and during other children’s programs (Article 10 of the Special Act on the Safety Management of Children’s Dietary Life, as amended 2010)
**Local Evidence**	
Law	The ***Advertisement Law of the People’s Republic of China*** states that advertisements claiming to replace mother’s milk with infant milk products, beverages, and other foods are prohibited. Using minors under ten years old as advertising spokespeople is also prohibited. Violations will result in penalties ranging from one to three times the advertising costs or fines ranging from CNY 100,000 to CNY 1,000,000 Yuan (USD 15,000 to USD 150,000).
Regulations (National)	The ***National Program for the Development of Children*** delineates stringent guidelines pertaining to child-centric food marketing. Broadcasts targeting children are prohibited from showcasing advertisements related to pharmaceuticals, health foods, alcoholic beverages, and especially those suggesting alternatives to breast milk. Emphasizing the well-being of children, the program restricts misleading and deleterious advertising content while bolstering efforts to penalize non-compliant practices. Furthermore, the regulations curtail children’s involvement in commercial promotional activities.
Industry Standards (Not mandatory)	In 2016, the China Advertising Association promulgated the “***Self-regulation Rules for Infant Formula Advertising***.” Central to its provisions, Article 5 mandates clear distinctions between imagery for and “older infant” formulas. Advertisements are prohibited from misrepresenting formula as analogous to breastmilk. To ensure compliance, the association enforces corrective advisories and, for recalcitrant or egregious offenders, issues public censures.
**Rating Score (Full points is 5)**	
**Suggestion**	*(To enhance the performance of this indicator, what specific recommendations do you propose for the future content and execution of related policies?)*

## Appendix B

**Table A2:** International Best Practice Examples (Benchmarks) on Food Marketing Indicator 1.

No.	Country	Content [10]
1.	Norway and Sweden	Per the Broadcasting Act, advertisements, irrespective of food content, are prohibited from being broadcast during or in association with children’s programs for those aged 12 and under.
2.	Quebec (Canada)	In Quebec, the only Canadian province to implement such measures since 1980, the Consumer Protection Act strictly proscribes commercial advertisements targeting children under 13 across various media. This determination considers the advert’s content, presentation, and broadcasting context. A threshold of 15% child viewership is set for protective measures against TV marketing. Violation of this statute at any commercial stage incurs significant penalties, ranging from CAD 600 to CAD 15,000 for individuals and CAD 2000 to CAD 100,000 for entities. Conversely, the rest of Canada relies on self-regulation, operationalized through the Canadian Children’s Food and Beverage Advertising Initiative (CAI) and enforced by Advertising Standards Canada (ASC) via The Broadcast Code for Advertising to Children.
3.	Chile	In 2012, Chile enacted the Law of Nutritional Composition of Food and Advertising (Ley 20,606), with implementation regulations ratified in June 2015 (Diario Oficia No 41.193). These stipulations demarcate ‘high’ content thresholds for calories, saturated fat, sugar, and sodium in foods and beverages. The legislation curtails advertising of ‘high in’ category foods directed at children under 14, defining such advertising based on both content and audience makeup, with a 20% child viewership benchmark. It also proscribes promotional tactics using cartoons, animations, or toys enticing children. As a notable instance of this regulation’s reach, items like Kinder Surprise eggs and toys in McDonald’s ‘Happy Meals’ were prohibited, with enforcement commencing on 1 July 2016.
4.	Ireland	In line with the 2013 revision of the Children’s Commercial Communications Code, advertisements of foods high in fats, sugars, and salt—as determined by a nutrient profiling model—are restricted during children’s TV and radio broadcasts with over 50% under-18 audience. Additionally, these foods are capped at 25% of total advertising time, with one in every four advertisements permissible. Any remaining adverts aimed at those below 13 years cannot employ health claims or licensed characters.
5.	South Korea	TV advertising to children less than 18 years of age is prohibited for specific categories of food before, during, and after programs shown between 5 and 7 pm and during other children’s programs (Article 10 of the Special Act on the Safety Management of Children’s Dietary Life, as amended 2010)

## Appendix C

**Table A3:** International Best Practice Examples (Benchmarks) on Food Marketing Indicator 2.

No.	Country	Content [10]
1.	Quebec (Canada)	In Quebec, the only Canadian province to implement such measures since 1980, the Consumer Protection Act strictly proscribes commercial advertisements targeting children under 13 across various media. This determination considers the advert’s content, presentation, and broadcasting context. A threshold of 15% child viewership initiates protective measures against TV marketing. Violation of this statute at any commercial stage incurs significant penalties, ranging from CAD 600 to CAD 15,000 for individuals and CAD 2000 to CAD 100,000 for entities. Conversely, the rest of Canada relies on self-regulation, operationalized through the Canadian Children’s Food and Beverage Advertising Initiative (CAI) and enforced by Advertising Standards Canada (ASC) via The Broadcast Code for Advertising to Children.
2.	Chile	In 2012, the government sanctioned the Law of Nutritional Composition of Food and Advertising (Ley 20,606). Subsequently, in June 2015, regulatory norms were instituted (Diario Oficial No 41.193) that delineated caloric, saturated fat, sugar, and sodium thresholds designating foods and beverages as ‘high’. Targeted advertising for these products is restricted for children under 14, defined by media directed towards or capturing more than 20% child viewership. Furthermore, certain promotional tactics, such as animation and toy incentives, are proscribed. This legislation, effective from 1 July 2016, extends to all advertising platforms and notably includes the prohibition of toys in McDonald’s ‘Happy Meals’ and the sale of Kinder Surprise eggs in Chile.

## Appendix D

**Table A4:** International Best Practice Examples (Benchmarks) on Food Marketing Indicator 3.

No.	Country	Content [10]
1.	Chile	In 2012, Chile initiated the Law of Nutritional Composition of Food and Advertising (Ley 20,606). By June 2015, related regulatory norms were established (Diario Oficial No 41.193), stipulating thresholds for caloric, fat, sugar, and sodium contents in foods deemed ‘high’. The law curtails advertising aimed at children below 14 for such products within educational premises. Furthermore, the directive, effective from 1 July 2016, proscribes certain child-centric promotional tactics, including animations and toy incentives.
2.	Spain	In 2011, Spain’s Law on Nutrition and Food Safety (Ley 17/2011) mandated educational institutions, including kindergartens and schools, to be advertisement-free. By July 2015, joint guidelines for food promotion, nutritional education, and physical activity campaigns were devised by AECOSAN in collaboration with the Regional Health Authorities. Compliance with the law is overseen by AECOSAN and the respective Regional Education and Health Administrations.
3.	Uruguay	In 2013, Uruguay enacted Law No. 19.140, titled ‘Healthy foods in schools’. This legislation bans the promotion of foods and beverages not aligned with the nutritional criteria stipulated in Article 3 and detailed in the 2014 school nutrition guidelines by the Ministry of Health. The prohibition encompasses all advertising mediums—ranging from billboards to brand placements on school items—and includes sponsorships and giveaways on school grounds. The law was operationalized in 2015.
